# Manual versus Mechanical Delivery of High-Quality Cardiopulmonary Resuscitation on a River-Based Fire Rescue Boat

**DOI:** 10.1017/S1049023X22001042

**Published:** 2022-10

**Authors:** Martin A.C. Manoukian, Daniel J. Tancredi, Matthew T. Linvill, Elisabeth H. Wynia, Brianna Beaver, John S. Rose, Bryn E. Mumma

**Affiliations:** 1. Department of Emergency Medicine, UC Davis, Sacramento, California USA; 2. Department of Pediatrics, UC Davis, Sacramento, California USA

**Keywords:** cardiac arrest, high-quality cardiopulmonary resuscitation, mechanical CPR, prehospital care, river-based CPR

## Abstract

**Objectives:**

Studies have demonstrated the efficacy of mechanical devices at delivering high-quality cardiopulmonary resuscitation (HQ-CPR) in various transport settings. Herein, this study investigates the efficacy of manual and mechanical HQ-CPR delivery on a fire rescue boat.

**Methods:**

A total of 15 active firefighter-paramedics were recruited for a prospective manikin-based trial. Each paramedic performed two minutes manual compression-only CPR while navigating on a river-based fire rescue boat. The boat was piloted in either a stable linear manner or dynamic S-turn manner to simulate obstacle avoidance. For each session of manual HQ-CPR, a session of mechanical HQ-CPR was also performed with a LUCAS 3 (Stryker; Kalamazoo, Michigan USA). A total of 60 sessions were completed. Parameters recorded included compression fraction (CF) and the percentage of compressions with correct depth >5cm (D%), correct rate 100-120 (R%), full release (FR%), and correct hand position (HP%). A composite HQ-CPR score was calculated as follows: ((D% + R% + FR% + HP%)/4) * CF%). Differences in magnitude of change seen in stable versus dynamic navigation within study conditions were evaluated with a Z-score calculation. Difficulty of HQ-CPR delivery was assessed utilizing the Borg Rating of Perceived Exertion Scale.

**Results:**

Participants were mostly male and had a median experience of 20 years. Manual HQ-CPR delivered during stable navigation out-performed manual HQ-CPR delivered during dynamic navigation for composite score and trended towards superiority for FR% and R%. There was no difference seen for any measured variable when comparing mechanical HQ-CPR delivered during stable navigation versus dynamic navigation. Mechanical HQ-CPR out-performed manual HQ-CPR during both stable and dynamic navigation in terms of composite score, FR%, and R%. Z-score calculation demonstrated that manual HQ-CPR delivery was significantly more affected by drive style than mechanical HQ-CPR delivery in terms of composite HQ-CPR score and trended towards significance for FR% and R%. Borg Rating of Perceived Exertion was higher for manual CPR delivered during dynamic sessions than for stable sessions.

**Conclusion:**

Mechanical HQ-CPR delivery is superior to manual HQ-CPR delivery during both stable and dynamic riverine navigation. Whereas manual HQ-CPR delivery was worse during dynamic transportation conditions compared to stable transport conditions, mechanical HQ-CPR delivery was unaffected by drive style. This suggests the utility of routine use of mechanical HQ-CPR devices in the riverine patient transport setting.

## Introduction

Out-of-hospital cardiac arrest (OHCA) affects millions of people every year globally with high rates of mortality and long-term morbidity.^1^ Along with rapid cardiac defibrillation in shockable rhythms, rapid administration of high-quality cardiopulmonary resuscitation (HQ-CPR) improves outcomes in OHCA.^2,3^ According to the American Heart Association (AHA; Dallas, Texas USA), HQ-CPR compressions necessitate a minimization of interruptions, a rate of 100-120, a depth >5 cm, and an adequate chest recoil.^1,4^ Mechanical CPR devices have been proposed as alternatives to manual CPR as a way to deliver reliable HQ-CPR while utilizing fewer human resources and preventing in-transport provider injury.^5,6^ Mechanical CPR devices have also been suggested to be efficacious in various transportation modalities, including ambulance, helicopter, and snow sled patient transport.^6–8^

In simulation studies, HQ-CPR performance is negatively affected by boat speed and ocean conditions in trained and lay personnel, causing up to a 30% decrease in effective compression delivery.^9–11^ Importantly in these studies, the boats were piloted in a linear course without turning. In real-life scenarios, HQ-CPR on a moving waterway vessel is likely affected by the vessel’s characteristics as well as the various forces that influence vessel displacement such as wind, waterway conditions, and waterway traffic. The effect of this displacement on the efficacy of manual HQ-CPR delivery has not been investigated. Additionally, the efficacy of mechanical delivery of HQ-CPR in the riverine rescue setting has not been investigated. As mechanical CPR machines are strapped to the patient, they may yield more consistent results that are less perturbed by the displacement experienced by a watercraft in transit.

In this study, the authors’ aimed to accomplish two goals. First, they sought to evaluate the effect of navigation style on HQ-CPR delivery by both human providers and a mechanical CPR device while aboard a moving small inland watercraft. Second, they sought to directly compare HQ-CPR delivered manually by human providers to HQ-CPR delivered by a mechanical device under these differing navigation conditions.

## Methods

### Study Design

After receiving an exemption from the UC Davis Institutional Review Board Administration (Sacramento, California USA; identification number 1656328-1), active firefighter-paramedics with water-based rescue experience were recruited from local agencies for a prospective manikin-based trial. Participant demographic information that was recorded included age, weight, height, sex, and years of paramedic experience.

**Figure 1.**
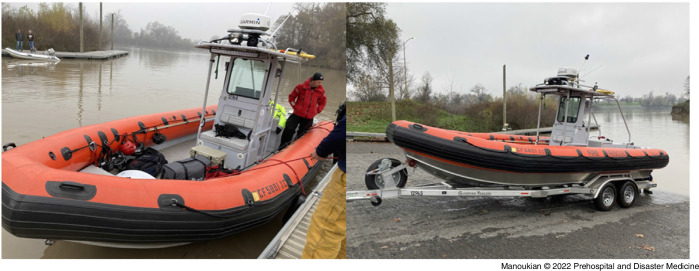
Rigid Hull Inflatable Vessel Used in Study.

**Figure 2.**
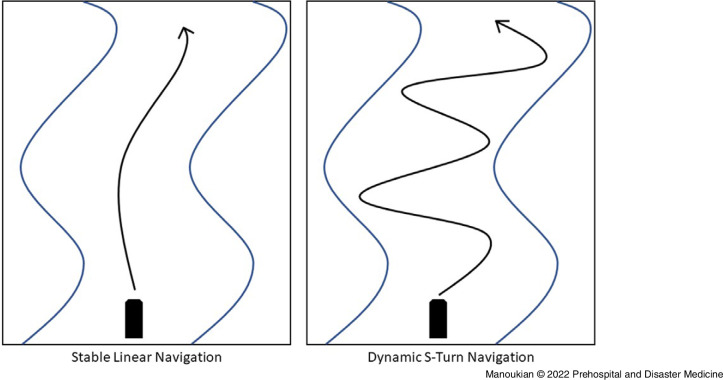
Representative Schematic of Drive Course Undertaken in Stable Linear Study Conditions and Dynamic S-Turn Study Conditions.

Manual compression-only HQ-CPR was performed on a Resusci-Anne manikin (Laerdal; Stavanger, Norway) while navigating on a 22-foot rigid hull inflatable rescue boat (Rogue Jet; White City, Oregon USA; Figure 1). Data were collected on the American River and Sacramento River (California USA) during periods of calm weather without concurrent boating traffic (Table 1).^12^ The vessel was operated at a target velocity of approximately 13.4 meters/second (30 miles/hour) in either a stable linear manner or dynamic S-turn manner to simulate obstacle avoidance (Figure 2). Each participant completed a two-minute session of compression-only HQ-CPR in the customary water rescue kneeling position during both stable and dynamic navigation sessions. Participants were randomized as to whether they performed HQ-CPR during stable navigation or dynamic navigation first. For each session of manual CPR, a separate independent session of mechanical HQ-CPR was also performed with a LUCAS 3 device (Stryker; Kalamazoo, Michigan USA). The performance of manual or mechanical HQ-CPR administration was alternated every two evolutions. The LUCAS device was re-applied to the manikin prior to each individual mechanical HQ-CPR session. A total of 60 two-minute HQ-CPR sessions were completed.

**Table 1.** Dates, Locations, and Weather Conditions during Data Acquisition

Meteorologic Data During Data Acquisition						
Date	Location	Temp (°F)	Wind Speed (Knots)	Humidity (%)	Barometer (Inch hg)	Visibility (Miles)
19-Oct 2021	38°36’05.7” N, 121°30’46.9” W	60 - 62	0 - 6	46 - 53	30	10
19-Oct 2021	38°36’05.7” N, 121°30’46.9” W	60 - 62	0 - 6	46 - 53	30	10
10-Dec 2021	38°41’40.8” N, 121°09’59.7” W	52 - 54	5 - 6	41-47	30.2	10
10-Dec 2021	38°41’40.8” N, 121°09’59.7” W	52 - 54	5 - 6	41-47	30.2	10
10-Dec 2021	38°41’40.8” N, 121°09’59.7” W	52 - 54	5 - 6	41-47	30.2	10
10-Dec 2021	38°41’40.8” N, 121°09’59.7” W	52 - 54	5 - 6	41-47	30.2	10
11-Dec 2021	38°41’40.8” N, 121°09’59.7” W	54 - 55	4 - 6	59 - 67	30.1	10
11-Dec 2021	38°41’40.8” N, 121°09’59.7” W	54 - 55	4 - 6	59 - 67	30.1	10
11-Dec 2021	38°41’40.8” N, 121°09’59.7” W	54 - 55	4 - 6	59 - 67	30.1	10
17-Dec 2021	38°41’40.8” N, 121°09’59.7” W	41 - 45	0 - 6	87 - 100	30.3	8 - 10
17-Dec 2021	38°41’40.8” N, 121°09’59.7” W	41 - 45	0 - 6	87 - 100	30.3	8 - 10
17-Dec 2021	38°41’40.8” N, 121°09’59.7” W	41 - 45	0 - 6	87 - 100	30.3	8 - 10
18-Dec 2021	38°36’05.7” N, 121°30’46.9” W	43 - 44	0 - 2.5	85 - 93	30.2	2
18-Dec 2021	38°36’05.7” N, 121°30’46.9” W	43 - 44	0 - 2.5	85 - 93	30.2	2
18-Dec 2021	38°36’05.7” N, 121°30’46.9” W	43 - 44	0 - 2.5	85 - 93	30.2	2

The HQ-CPR parameters were recorded utilizing the SkillReporter PC Application (Laerdal; Stavanger, Norway). Parameters recorded included compression fraction (CF%) and the percentage of compressions with correct depth >5cm (D%), correct rate 100-120 (R%), full release (FR%), and correct hand position (HP%). A composite HQ-CPR score was adapted from previous literature to incorporate all Level 1 or Level 2a recommendations from the AHA and was calculated as follows: ((D% + R% + FR% + HP%)/4) * CF%).^9,10^ A compression duty cycle percentage was also recorded. Difficulty of HQ-CPR delivery was assessed utilizing a questionnaire which included the Borg Rating of Perceived Exertion Scale.^13^

Mean, median, standard deviation, and interquartile ranges of HQ-CPR parameters were calculated for each study condition. A two tailed paired t-test as well as a change score and 95% confidence interval were calculated to evaluate for statistical differences in HQ-CPR outcomes between the manual stable and manual dynamic study conditions. A two tailed heteroscedastic t-test and 95% confidence intervals were calculated to evaluate for statistical differences in HQ-CPR outcomes between the mechanical stable versus mechanical dynamic conditions, manual stable versus mechanical stable conditions, and manual dynamic versus mechanical dynamic conditions. A Z-score was calculated to evaluate the difference in the magnitude of change seen in the manual stable versus manual dynamic conditions compared to the change seen in the mechanical stable versus mechanical dynamic conditions. Difficulty of manual HQ-CPR delivery during stable and dynamic sessions was compared using a paired two tailed student’s t-test.

## Results

A total of 15 participants were recruited for this study. All participants were active-duty firefighter-paramedics with Advanced Cardiac Life Support/ACLS certification. Participant demographics can be seen in Table 2. Participants were overwhelmingly male and had a median of 20 (25^th^, 75^th^ percentile: 7.5, 23) years of Emergency Medical Service experience.

**Table 2.** Participant Demographics Presented as Percentage or Median (25^th^-75^th^ Percentile)

Participant Demographics (n = 15)	
Male Gender	14 (93%)
Age (Years)	39 (32.5 – 48)
Height (cm)	180.34 (177.80 – 185.42)
Weight (kg)	92.98 (76.88 – 96.66)
Experience (Years)	20 (7.5 – 23)

**Figure 3.**
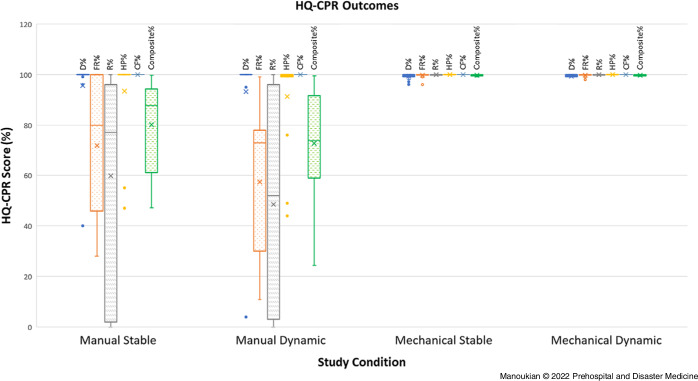
Box and Whisker Plot of HQ-CPR Outcomes in the Four Study Conditions for Various HQ-CPR Measurements. Abbreviations: D%, percentage of compressions with correct depth >5cm; FR%, percentage of compressions with full chest release; R%, percentage of compressions with correct rate 100-120 compressions per minute; HP%, percentage of compressions with correct hand position; CF%, compression fraction; Composite %, final composite score as calculated by the equation ((D% + R% + FR% + HP%)/4) * CF%); HQ-CPR, high-quality cardiopulmonary resuscitation.

The HQ-CPR results from the four study conditions can be seen in Table 3 and Figure 3. All four study conditions achieved a CF% of 100%. Manual HQ-CPR delivered during stable navigation was statistically superior to manual HQ-CPR delivered during dynamic navigation only in terms of composite HQ-CPR score, however it trended towards superiority in terms of FR% and R%. There was no statistical difference seen in any HQ-CPR parameter when comparing mechanical HQ-CPR delivered during stable versus dynamic navigation. Mechanical HQ-CPR out-performed manual HQ-CPR during both stable and dynamic navigation in terms of FR%, R%, and composite HQ-CPR score. Mechanical HQ-CPR trended towards superior HP% in both stable and dynamic navigation groups, however did not reach statistical significance. Additionally, mechanical HQ-CPR averaged a slower rate, a lower depth of compression, and a longer duty cycle than manual HQ-CPR during both stable and dynamic navigation.

**Table 3.** HQ-CPR Outcomes in the Four Study Conditions Reported as Average (SD) and Differences between Study Conditions Reported in Mean Difference (95% CI)

HQ-CPR Outcomes in the Four Study Conditions				
	Manual Stable Average (Standard Deviation)		Manual Dynamic Average (Standard Deviation)	
Depth %	95.7	(15.43)	93.3	(24.73)
Full Recoil %	71.8	(26.96)	57.5	(30.00)
Rate %	62.7	(46.25)	48.7	(41.14)
Compression Fraction %	100.0	(0.00)	100.0	(0.00)
Hand Positioning %	93.5	(17.31)	91.2	(19.18)
Composite HQ-CPR Score	80.9	(17.51)	72.7	(20.22)
Average Depth (mm)	61.3	(4.08)	60.1	(6.98)
Average Rate (Compressions/Second)	119.2	(12.66)	119.3	(13.13)
Duty Cycle	39.5	(5.87)	39.4	(5.49)
	**Mechanical Stable** **Average (Standard Deviation)**		**Mechanical Dynamic** **Average (Standard Deviation)**	
Depth %	99.2	(1.26)	99.3	(1.22)
Full Recoil %	99.7	(1.05)	99.8	(0.56)
Rate %	100.0	(0.00)	100.0	(0.00)
Compression Fraction %	100.0	(0.00)	100.0	(0.00)
Hand Positioning %	100.0	(0.00)	100.0	(0.00)
Composite HQ-CPR Score	99.7	(0.36)	99.8	(0.31)
Average Depth (mm)	54.3	(1.40)	54.5	(0.92)
Average Rate (Compressions/Minute)	111.0	(0.00)	111.0	(0.00)
Duty Cycle	53.1	(2.02)	52.9	(0.96)
**Differences in HQ-CPR Outcomes Between the Four Study Conditions**				
	**Manual Stable** **vs** **Manual Dynamic** **Difference (95% CI)**	**Mechanical Stable** **vs** **Mechanical Dynamic** **Difference (95% CI)**	**Manual Stable** **vs** **Mechanical Stable** **Difference (95% CI)**	**Manual Dynamic** **vs** **Mechanical Dynamic** **Difference (95% CI)**
Depth %	−2.4 (−7.6 – 2.8) P = .33	−0.07 (−1.0 – 0.9) P = .88	3.5 (−4.6 – 11.7) P = .39	6.00 (−7.1 – 19.1) P = .36
Full Recoil %	−14.3 (−31.1 – 2.5) P = .09	−0.1 (−0.8 – 0.5) P = .67	**27.9 (13.6 – 42.1)** **P = .001**	**42.3 (26.7 – 58.2)** **P <.001**
Rate %	−11.1 (−26.2 – 3.9) P = .14	–	**40.2 (17.1 – 63.3)** **P = .003**	**51.33 (29.6 – 73.1)** **P <.001**
Compression Fraction %	–	–	–	–
Hand Positioning %	−2.3 (−5.7 – 1.2) P = .18	–	6.5 (−2.6 – 15.7) P = .17	8.8 (−1.3 – 18.9) P = .10
Composite HQ-CPR Score %	**-7.5 (−14.7 – −0.3)** **P = .04**	−0.05 (−0.3 – 0.23) P = .69	**19.5 (10.6 – 28.5)** **P < .001**	**27.1 (16.4 – 37.8)** **P <.001**
Average Depth (mm)	−1.1 (−31 – 0.8) P = .23	−0.2 (−1.1 – 0.7) P = .65	**6.9 (4.7 – 9.2)** **P <.001**	**5.9 (1.9 – 9.3)** **P = .008**
Average Rate (Compressions/Minute)	0.07 (−4.9 – 5.0) P = .98	–	**8.2 (1.5 – 14.6)** **P = .025**	**8.3 (1.3 – 15.2)** **P = .029**
Duty Cycle	−0.07 (−1.4 – 1.2) P = .92	0.13 (−1.0 – 1.3) P = .96	**13.6 (10.3 – 16.9)** **P <.001**	**13.5 (10.6 – 16.5)** **P <.001**

Abbreviation: HQ-CPR, high-quality cardiopulmonary resuscitation.

Z-score calculations can be seen in Table 4. This evaluation revealed a significant difference in the magnitude of change seen between manual stable versus manual dynamic conditions compared to mechanical stable versus mechanical dynamic conditions regarding composite score. Differences in the FR% score approached but did not reach statistical significance. Differences in the R% score also trended towards but did not reach significance, however within group standard deviations for this calculation included a value of 0 and so should be interpreted with caution.

**Table 4.** Z-Score Calculation to Evaluate for a Difference in the Magnitude of Change Seen in the Manual Stable vs Manual Dynamic Conditions Compared to the Mechanical Stable vs Mechanical Dynamic Conditions

Evaluation for Difference in Magnitude of Change Seen in the Manual Stable vs Manual Dynamic Conditions Compared to the Mechanical Stable vs Mechanical Dynamic Conditions		
HQ-CPR Variable Score	Difference in Magnitude Seen in Manual and Mechanical Study Arms	Z-Test P Value
Depth %	2.45 (−2.3 – 7.3)	.31
Recoil %	14.5 (−0.9 – 24.9)	.065
Rate %	11.1 (−2.65 – 24.9)	.11^a^
Compression Fraction %	0	–
Hand Positioning %	2.3 (−0.89 – 5.42)	.16^a^
Composite Score %	7.6 (1.0 – 14.2)	.024
Average Depth (cm)	1.3 (−0.6 – 3.31)	.19
Average Rate (Compressions per Minute)	−0.07 (−4.6 – 4.5)	.98^a^
Duty Cycle	−0.07 (−1.7 – 1.6)	.94

Note: Values are reported as mean difference (95% confidence interval).

a Within group standard deviation for the evaluation of the mechanical stable vs mechanical dynamic conditions was 0, which may affect validity of Z-score.

Borg Rating of Perceived Exertion among participants was significantly higher for manual HQ-CPR delivery during dynamic navigation compared to manual HQ-CPR delivery during stable navigation (4.93 [SD = 1.33] versus 2.87 [SD = 0.92]; P <.001). When asked to compare the difficulty of HQ-CPR delivery in a small waterway vessel during stable navigation with performing HQ-CPR in a moving ambulance, eight (53%) participants responded that it was the same difficulty level and four (27%) participants responded that it was somewhat more difficult or much more difficult. When asked to compare the difficulty of HQ-CPR delivery in a small waterway vessel during dynamic navigation to performing HQ-CPR in a moving ambulance, two (13%) participants responded that it was the same difficulty level and thirteen (87%) participants responded that it was somewhat more difficult or much more difficult.

## Discussion

This study investigated the effectiveness of manual and mechanical delivery of HQ-CPR chest compressions in a small waterway rescue vessel. Navigation and the delivery of HQ-CPR during transport in a riverine setting differs dramatically from land-based transport due to differences that include vehicle structure, braking systems, transportation medium (water versus asphalt), and the presence of an underlying current. Previous studies have demonstrated that increasing vessel speed, rough waters, and provider fatigue contributed to poor HQ-CPR outcomes during maritime transport.^9–11^ Though this study was performed during calm weather conditions, the vessel was piloted in two separate patterns: a stable pattern to simulate transportation in calm, unobstructed waterways as well as in a dynamic S-turn pattern to simulate avoidance of obstacles and boat displacement during more difficult conditions. Examples of obstacles that may be encountered during riverine patient transport include recreational boaters, kayakers, paddle boarders, swimmers, floating debris, or static geologic and botanical features.

In this study, manual HQ-CPR delivered during stable navigation was significantly better than manual HQ-CPR delivered during dynamic navigation. In particular, FR% and R% trended negatively in the dynamic arm, though did not reach statistical significance. These results likely reflect an increase in the amount of leaning required to maintain balance during navigation. The off-balancing forces experienced during dynamic navigation were likely higher than those experienced during stable navigation, requiring the use of intermittent tripoding by the participants. This likely reduced the amount of full chest recoil experienced by the manikin as well as altered the delivery rate of chest compressions. The use of a metronome has been shown to improve compression rate during the delivery of HQ-CPR, however noise pollution from the wind and boat engine made metronome use in this study impractical.^14^

Previous studies have suggested that fatigue during maritime HQ-CPR delivery is largely due to local fatigue of postural muscles rather than aerobic metabolic demand.^11^ In the current study, dynamic navigation resulted in a higher rating of perceived exertion and likely contributed to the lower HQ-CPR scores. Importantly, HQ-CPR in this study was delivered with the providers in the customary water rescue kneeling position. This has been shown to improve the quality of HQ-CPR delivery and may have served to reduce but not eliminate the effects of postural fatigue.^15^ Despite this fact, participants still found HQ-CPR delivery during dynamic navigation to be more difficult than their standard HQ-CPR delivery in a moving ambulance. Additionally, the participants in this study were firefighter-paramedics with rigorous physical fitness standards. Thus, the rating of perceived exertion during dynamic navigation may be higher in participant populations with lesser physical conditioning.

In contrast to the manual HQ-CPR delivery, the efficacy of mechanical HQ-CPR delivery was unaffected by navigation style. There was no significant difference in any of the recorded HQ-CPR parameters seen between the stable mechanical HQ-CPR and dynamic mechanical HQ-CPR study conditions. Additionally, Z-score evaluation demonstrated significantly less change in composite HQ-CPR score during different navigation styles for mechanical HQ-CPR delivery compared to manual HQ-CPR delivery and trended towards significance for FR% and R%. This suggests that mechanical HQ-CPR delivery is much less affected by increased off-balancing forces and could be valuable when it is necessary to deliver HQ-CPR in scenarios that increase displacement of the vessel during transport.

Mechanical HQ-CPR out-performed manual HQ-CPR in terms of FR%, R%, and composite HQ-CPR score for stable and dynamic evolutions and trended towards better HP% for both types of drive style. Though there was no difference in D% between manual and mechanical HQ-CPR for stable or dynamic navigation, mechanical HQ-CPR averaged a shallower depth than manual HQ-CPR, achieving a mean depth within the 5-6cm depth range suggested by the AHA.^16^ Mechanical HQ-CPR also resulted in a lower average compression rate with significantly less variability for both stable and dynamic evolutions. For both stable and dynamic navigation, manual HQ-CPR had a duty cycle of approximately 39.4%, which is similar to previous studies.^17^ In contrast, mechanical HQ-CPR achieved a duty cycle of approximately 53%, closer to the 50% goal recommended by the AHA, although this is only a Level 2b recommendation with Level C quality of evidence.^16^

Despite its demonstrated effectiveness in a variety of simulated scenarios, mechanical HQ-CPR has yet to demonstrate a benefit over manual HQ-CPR in prospective randomized trials in terms of patient outcomes.^18–20^ It may be that a more targeted approach to the use of mechanical HQ-CPR devices would yield superior results rather than universal deployment. Scenarios that limit provider access to the patient such as helicopter transport or prolonged gurney transport in narrow passages have been suggested to be more appropriate for mechanical HQ-CPR use.^21–23^ Like helicopters, small waterway vessels afford only restricted space for providers to operate, limiting the speed and reliability with which procedures, including HQ-CPR, can be performed. Transportation in a small water rescue vessel also necessitates multiple episodes of relocation, such as from the initial contact point into the vessel, from the vessel into an ambulance, and from the ambulance to the final point of care. Future research investigating HQ-CPR delivery during a prolonged transport and relocation periods within the riverine setting may further elucidate the benefits of mechanical HQ-CPR delivery. A factor of riverine patient transport that may negatively affect the efficacy of mechanical HQ-CPR is moisture on the patient’s chest, which may increase slippage of the device during compression cycles.

## Limitations

Limitations include the limited number of evolutions performed, the omission of ventilations, omission of rhythm checks, a manikin-based approach rather than real-life scenarios, and the performance of only two minutes of CPR, which would likely mitigate fatigue factor and hands-off time. This study also failed to evaluate the effects of patient movement into or out of the rescue vessel and did not account for moisture which may be present in a river-based rescue scenario.

## Conclusion

This study suggests the superiority of mechanical HQ-CPR delivery over manual HQ-CPR delivery during patient transport in a riverine setting. Whereas manual HQ-CPR delivery was negatively affected during dynamic transportation conditions, mechanical HQ-CPR delivery was unaffected. This suggests the utility of routine use of mechanical HQ-CPR devices in the riverine patient transport setting, though further investigation is necessary.
